# Supplementary material for: Dynamics of the hindgut microbiota of the Japanese honey bees (Apis cerana japonica) throughout the overwintering period
Source: PeerJ. 2025 Oct 13;13:e20050. doi: 10.7717/peerj.20050 (PMC12530201; doi:10.7717/peerj.20050)
Supplement: Supplemental Information 1 [file peerj-13-20050-s001.docx]

Supplementary information for

**Dynamics of the hindgut microbiota of the Japanese honey bees (*Apis cerana japonica*) throughout the overwintering period**

Akihiko Suzuki ^1^, Shumpei Hisamoto ^2^, Yoshiko Sakamoto ^1^

^1^ *National Institute for Environmental Studies, Tsukuba, Ibaraki, 305-8506, Japan*

^2^ *Meiji Institute for Advanced Study of Mathematical Sciences, Meiji University, Nakano, Tokyo, 164-8525, Japan*

**Corresponding author**: Akihiko Suzuki

*National Institute for Environmental Studies, Tsukuba, Ibaraki, 305-8506, Japan*

E-mail: brak13072@gmail.com, suzuki.akihiko@nies.go.jp

Tel: +81-(0)29-850-2480

| Sample | Period^1)^ | input | filtered | denoisedF | denoisedR | merged | nonchim |
| --- | --- | --- | --- | --- | --- | --- | --- |
| H10-1 | BO | 40,227 | 35,416 | 35,395 | 35,411 | 35,243 | 32,425 |
| H10-2 | BO | 34,079 | 30,018 | 29,992 | 30,008 | 29,646 | 27,822 |
| H10-3 | BO | 34,002 | 30,516 | 30,487 | 30,507 | 30,249 | 27,766 |
| H12-1 | OW | 40,257 | 35,471 | 35,454 | 35,465 | 35,304 | 31,643 |
| H12-2 | OW | 40,056 | 35,092 | 35,050 | 35,076 | 34,918 | 32,405 |
| H12-3 | OW | 46,498 | 40,919 | 40,879 | 40,905 | 40,701 | 37,821 |
| H3-1 | AO | 32,360 | 28,886 | 28,856 | 28,886 | 28,762 | 26,728 |
| H3-2 | AO | 26,409 | 23,369 | 23,345 | 23,366 | 23,316 | 22,433 |
| H3-3 | AO | 29,966 | 26,708 | 26,683 | 26,703 | 26,572 | 25,611 |
| I10-1 | BO | 40,468 | 36,072 | 36,001 | 36,059 | 35,894 | 32,594 |
| I10-2 | BO | 38,863 | 34,281 | 34,226 | 34,266 | 34,076 | 30,863 |
| I10-3 | BO | 39,201 | 34,580 | 34,544 | 34,576 | 34,444 | 31,541 |
| I12-1 | OW | 39,002 | 34,621 | 34,553 | 34,548 | 34,222 | 31,527 |
| I12-2 | OW | 40,961 | 36,421 | 36,360 | 36,397 | 35,785 | 31,655 |
| I12-3 | OW | 38,175 | 34,027 | 33,973 | 34,002 | 33,377 | 29,494 |
| I3-1 | AO | 34,664 | 30,664 | 30,578 | 30,628 | 30,348 | 27,388 |
| I3-2 | AO | 38,045 | 33,657 | 33,600 | 33,641 | 33,483 | 30,721 |
| I3-3 | AO | 45,948 | 40,630 | 40,461 | 40,563 | 40,180 | 36,105 |
| T10-1 | BO | 43,493 | 38,331 | 38,302 | 38,314 | 37,899 | 32,305 |
| T10-2 | BO | 37,892 | 33,190 | 33,140 | 33,173 | 32,854 | 27,484 |
| T10-3 | BO | 42,628 | 37,660 | 37,618 | 37,637 | 37,346 | 32,907 |
| T12-1 | OW | 49,821 | 43,801 | 43,743 | 43,784 | 43,481 | 38,080 |
| T12-2 | OW | 38,125 | 33,463 | 33,423 | 33,453 | 33,216 | 28,936 |
| T12-3 | OW | 34,798 | 30,556 | 30,510 | 30,547 | 30,299 | 26,394 |
| T3-1 | AO | 39,387 | 34,797 | 34,736 | 34,779 | 34,547 | 32,241 |
| T3-2 | AO | 34,039 | 30,128 | 30,076 | 30,119 | 30,011 | 28,665 |
| T3-3 | AO | 34,069 | 30,210 | 30,174 | 30,191 | 29,929 | 28,321 |
| X10-1 | BO | 37,821 | 33,753 | 33,713 | 33,739 | 33,499 | 28,812 |
| X10-2 | BO | 39,593 | 35,442 | 35,409 | 35,430 | 35,279 | 31,784 |
| X10-3 | BO | 47,492 | 42,486 | 42,454 | 42,473 | 42,094 | 36,332 |
| X12-1 | OW | 36,862 | 32,927 | 32,906 | 32,921 | 32,830 | 31,203 |
| X12-2 | OW | 43,581 | 38,825 | 38,773 | 38,808 | 38,611 | 34,540 |
| X12-3 | OW | 29,934 | 26,880 | 26,862 | 26,877 | 26,811 | 25,485 |
| X3-1 | AO | 33,250 | 29,117 | 29,063 | 29,104 | 28,877 | 26,721 |
| X3-2 | AO | 44,902 | 39,275 | 39,170 | 39,224 | 38,748 | 35,553 |
| X3-3 | AO | 34,158 | 29,749 | 29,711 | 29,736 | 29,571 | 26,384 |

**Table S1**. Number of reads obtained after filtering and trimming each sample using DADA2.

^1)^ Before overwintering: BO, during overwintering: OW, after overwintering: AO.
The alphabet in the sample indicates the colony ID, and the subsequent number indicates the month the sample was collected.

**Table S2**. Number of coverages after rarefaction at the minimum lead (21,808) for each sample.

| Sample | Period^1)^ | Colony | Coverage |
| --- | --- | --- | --- |
| H10-1 | BO | H | 0.99954 |
| H10-2 | BO | H | 0.99977 |
| H10-3 | BO | H | 0.99959 |
| H12-1 | OW | H | 0.99963 |
| H12-2 | OW | H | 0.99977 |
| H12-3 | OW | H | 0.99945 |
| H3-1 | AO | H | 0.99950 |
| H3-2 | AO | H | 0.99959 |
| H3-3 | AO | H | 0.99963 |
| I10-1 | BO | I | 0.99950 |
| I10-2 | BO | I | 0.99954 |
| I10-3 | BO | I | 0.99945 |
| I12-1 | OW | I | 0.99945 |
| I12-2 | OW | I | 0.99945 |
| I12-3 | OW | I | 0.99950 |
| I3-1 | AO | I | 0.99954 |
| I3-2 | AO | I | 0.99940 |
| I3-3 | AO | I | 0.99972 |
| T10-1 | BO | T | 0.99982 |
| T10-2 | BO | T | 0.99917 |
| T10-3 | BO | T | 0.99950 |
| T12-1 | OW | T | 0.99931 |
| T12-2 | OW | T | 0.99954 |
| T12-3 | OW | T | 0.99936 |
| T3-1 | AO | T | 0.99922 |
| T3-2 | AO | T | 0.99959 |
| T3-3 | AO | T | 0.99963 |
| X10-1 | BO | X | 0.99950 |
| X10-2 | BO | X | 0.99936 |
| X10-3 | BO | X | 0.99950 |
| X12-1 | OW | X | 0.99954 |
| X12-2 | OW | X | 0.99945 |
| X12-3 | OW | X | 0.99968 |
| X3-1 | AO | X | 0.99950 |
| X3-2 | AO | X | 0.99945 |
| X3-3 | AO | X | 0.99950 |

^1)^ Before overwintering: BO, during overwintering: OW, after overwintering: AO

The alphabet in the sample indicates the colony ID, and the subsequent number indicates the month the sample was collected.

**Table S3**. Relative abundance of bacterial phyla in the hindgut microbiota of the Japanese honey bees (*Apis cerana japonica*) in all samples.

| Phylum | n | Minimum | Maximum | Mean | Standard deviation |
| --- | --- | --- | --- | --- | --- |
| Actinobacteriota | 36 | 0.40% | 13.10% | 3.60% | 2.60% |
| Bacteroidota | 36 | 11.70% | 36.30% | 21.00% | 6.20% |
| Firmicutes | 36 | 9.20% | 33.90% | 19.20% | 6.30% |
| Proteobacteria | 36 | 35.20% | 68.60% | 56.20% | 7.20% |
| Others | 36 | 0.00% | 0.50% | 0.10% | 0.10% |

**Table S4**. Relative abundance of bacterial phyla in the hindgut microbiota of the Japanese honey bees (*Apis cerana japonica*) at each of the three sampling periods.

| Phylum | Period^1)^ | n | Minimum | Maximum | Mean | Standard deviation |
| --- | --- | --- | --- | --- | --- | --- |
| Actinobacteriota | BO | 12 | 0.40% | 4.40% | 2.10% | 1.30% |
| Actinobacteriota | OW | 12 | 0.70% | 13.10% | 5.20% | 3.00% |
| Actinobacteriota | AO | 12 | 0.80% | 9.40% | 3.40% | 2.50% |
| Bacteroidota | BO | 12 | 16.40% | 30.80% | 22.50% | 4.50% |
| Bacteroidota | OW | 12 | 12.70% | 35.10% | 19.60% | 6.60% |
| Bacteroidota | AO | 12 | 11.70% | 36.30% | 20.90% | 7.30% |
| Firmicutes | BO | 12 | 9.20% | 21.20% | 15.40% | 4.40% |
| Firmicutes | OW | 12 | 13.10% | 33.00% | 22.80% | 6.00% |
| Firmicutes | AO | 12 | 10.50% | 33.90% | 19.40% | 6.30% |
| Proteobacteria | BO | 12 | 55.10% | 65.50% | 59.90% | 3.70% |
| Proteobacteria | OW | 12 | 41.10% | 62.00% | 52.30% | 6.20% |
| Proteobacteria | AO | 12 | 35.20% | 68.60% | 56.30% | 9.10% |
| Others | BO | 12 | 0.00% | 0.00% | 0.00% | 0.00% |
| Others | OW | 12 | 0.00% | 0.50% | 0.10% | 0.10% |
| Others | AO | 12 | 0.00% | 0.30% | 0.10% | 0.10% |

^1)^ Before overwintering: BO, during overwintering: OW, after overwintering: AO

**Table S5**. Relative abundance of bacterial genera in the hindgut microbiota of the Japanese honey bees (*Apis cerana japonica*) in all samples.

| Genus | n | Minimum | Maximum | Mean | Standard deviation |
| --- | --- | --- | --- | --- | --- |
| *Apibacter* | 36 | 11.60% | 36.30% | 21.00% | 6.20% |
| *Bifidobacterium* | 36 | 0.20% | 13.10% | 3.40% | 2.70% |
| *Bombilactobacillus* | 36 | 0.30% | 13.60% | 4.10% | 3.30% |
| *Gilliamella* | 36 | 26.60% | 59.80% | 47.10% | 8.80% |
| *Lactobacillus* | 36 | 6.90% | 28.20% | 14.90% | 4.80% |
| *Snodgrassella* | 36 | 0.70% | 22.40% | 7.30% | 6.30% |
| Others | 36 | 0.00% | 12.10% | 1.50% | 2.60% |
| Unclassified | 36 | 0.00% | 5.40% | 0.80% | 1.50% |

**Table S6**. Relative abundance of bacterial genera in the hindgut microbiota of the Japanese honey bees (*Apis cerana japonica*) at each of the three sampling periods.

| Genus | Period^1)^ | n | Minimum | Maximum | Mean | Standard deviation |
| --- | --- | --- | --- | --- | --- | --- |
| *Apibacter* | BO | 12 | 16.40% | 30.80% | 22.50% | 4.50% |
| *Apibacter* | OW | 12 | 12.70% | 35.10% | 19.60% | 6.60% |
| *Apibacter* | AO | 12 | 11.60% | 36.30% | 20.90% | 7.30% |
| *Bifidobacterium* | BO | 12 | 0.40% | 4.20% | 2.10% | 1.30% |
| *Bifidobacterium* | OW | 12 | 0.70% | 13.10% | 5.20% | 3.00% |
| *Bifidobacterium* | AO | 12 | 0.20% | 9.40% | 3.00% | 2.70% |
| *Bombilactobacillus* | BO | 12 | 0.70% | 6.60% | 2.20% | 1.70% |
| *Bombilactobacillus* | OW | 12 | 1.30% | 10.80% | 5.50% | 2.80% |
| *Bombilactobacillus* | AO | 12 | 0.30% | 13.60% | 4.60% | 4.10% |
| *Gilliamella* | BO | 12 | 38.20% | 59.80% | 48.80% | 7.60% |
| *Gilliamella* | OW | 12 | 26.60% | 57.30% | 44.30% | 10.50% |
| *Gilliamella* | AO | 12 | 31.40% | 57.60% | 48.10% | 8.10% |
| *Lactobacillus* | BO | 12 | 6.90% | 19.50% | 13.00% | 4.40% |
| *Lactobacillus* | OW | 12 | 10.60% | 28.20% | 17.40% | 5.20% |
| *Lactobacillus* | AO | 12 | 7.60% | 20.10% | 14.30% | 4.00% |
| *Snodgrassella* | BO | 12 | 0.70% | 22.40% | 10.80% | 9.10% |
| *Snodgrassella* | OW | 12 | 0.90% | 10.80% | 5.70% | 3.60% |
| *Snodgrassella* | AO | 12 | 1.20% | 11.10% | 5.30% | 3.10% |
| Others | BO | 12 | 0.00% | 1.80% | 0.60% | 0.60% |
| Others | OW | 12 | 0.10% | 12.10% | 1.30% | 3.40% |
| Others | AO | 12 | 0.50% | 10.10% | 2.60% | 2.60% |
| Unclassified | BO | 12 | 0.00% | 0.40% | 0.10% | 0.10% |
| Unclassified | OW | 12 | 0.00% | 4.40% | 1.10% | 1.90% |
| Unclassified | AO | 12 | 0.10% | 5.40% | 1.30% | 1.70% |

^1)^ Before overwintering: BO, during overwintering: OW, after overwintering: AO

**Table S7**. Results of pairwise permutational multivariate analysis of variance of the hindgut microbiota in the Japanese honey bees (*Apis cerana japonica*) from four colonies during three sampling periods.

| Group | Pair ^1)^ | Df | Sums Of Sqs | F. Model | R^2^ | p. adjusted |
| --- | --- | --- | --- | --- | --- | --- |
| Period^1)^ | BO vs. OW | 1 | 0.333 | 3.037 | 0.121 | 0.029 |
|  | BO vs. AO | 1 | 0.195 | 1.861 | 0.078 | 0.225 |
|  | OW vs. AO | 1 | 0.199 | 1.973 | 0.082 | 0.159 |

^1)^ Before overwintering: BO, during overwintering: OW, after overwintering: AO

**Table S8**. Generalized linear mixed model analysis. The number of reads of the six core bacterial genera in the hindgut microbiota of the Japanese honey bees (*Apis cerana japonica*) was used as the objective variable, the three time periods as explanatory variables, and the colony as a random effect. BO group is set as a reference group.

|  | Period ^1)^ | Estimate | Standard error | Z score | 95% Cl low | 95% Cl high | p value |
| --- | --- | --- | --- | --- | --- | --- | --- |
| *Apibacter* | BO | 8.492 | 0.065 | 130.221 | 8.364 | 8.620 | <0.0001 |
|  | OW | -0.159 | 0.142 | -1.118 | -0.437 | 0.120 | 0.264 |
|  | AO | -0.096 | 0.173 | -0.554 | -0.435 | 0.243 | 0.579 |
| *Bifidobacterium* | BO | 5.995 | 0.267 | 22.479 | 5.472 | 6.518 | <0.0001 |
|  | OW | 0.977 | 0.377 | 2.587 | 0.237 | 1.716 | 0.010 |
|  | AO | 0.286 | 0.421 | 0.679 | -0.540 | 1.112 | 0.497 |
| *Bombilactobacillus* | BO | 5.988 | 0.284 | 21.062 | 5.430 | 6.545 | <0.0001 |
|  | OW | 1.036 | 0.458 | 2.262 | 0.138 | 1.933 | 0.024 |
|  | AO | 0.657 | 0.628 | 1.046 | -0.574 | 1.889 | 0.296 |
| *Gilliamella* | BO | 9.264 | 0.064 | 145.277 | 9.139 | 9.389 | <0.0001 |
|  | OW | -0.107 | 0.159 | -0.676 | -0.419 | 0.204 | 0.499 |
|  | AO | -0.010 | 0.096 | -0.104 | -0.198 | 0.178 | 0.917 |
| *Lactobacillus* | BO | 7.908 | 0.135 | 58.672 | 7.644 | 8.173 | <0.0001 |
|  | OW | 0.320 | 0.096 | 3.321 | 0.131 | 0.509 | 0.001 |
|  | AO | 0.134 | 0.130 | 1.032 | -0.121 | 0.389 | 0.302 |
| *Snodgrassella* | BO | 7.199 | 0.619 | 11.633 | 5.986 | 8.412 | <0.0001 |
|  | OW | -0.269 | 0.834 | -0.322 | -1.903 | 1.365 | 0.747 |
|  | AO | -0.233 | 0.537 | -0.433 | -1.286 | 0.820 | 0.665 |

^1)^ Before overwintering: BO, during overwintering: OW, after overwintering: AO.

**
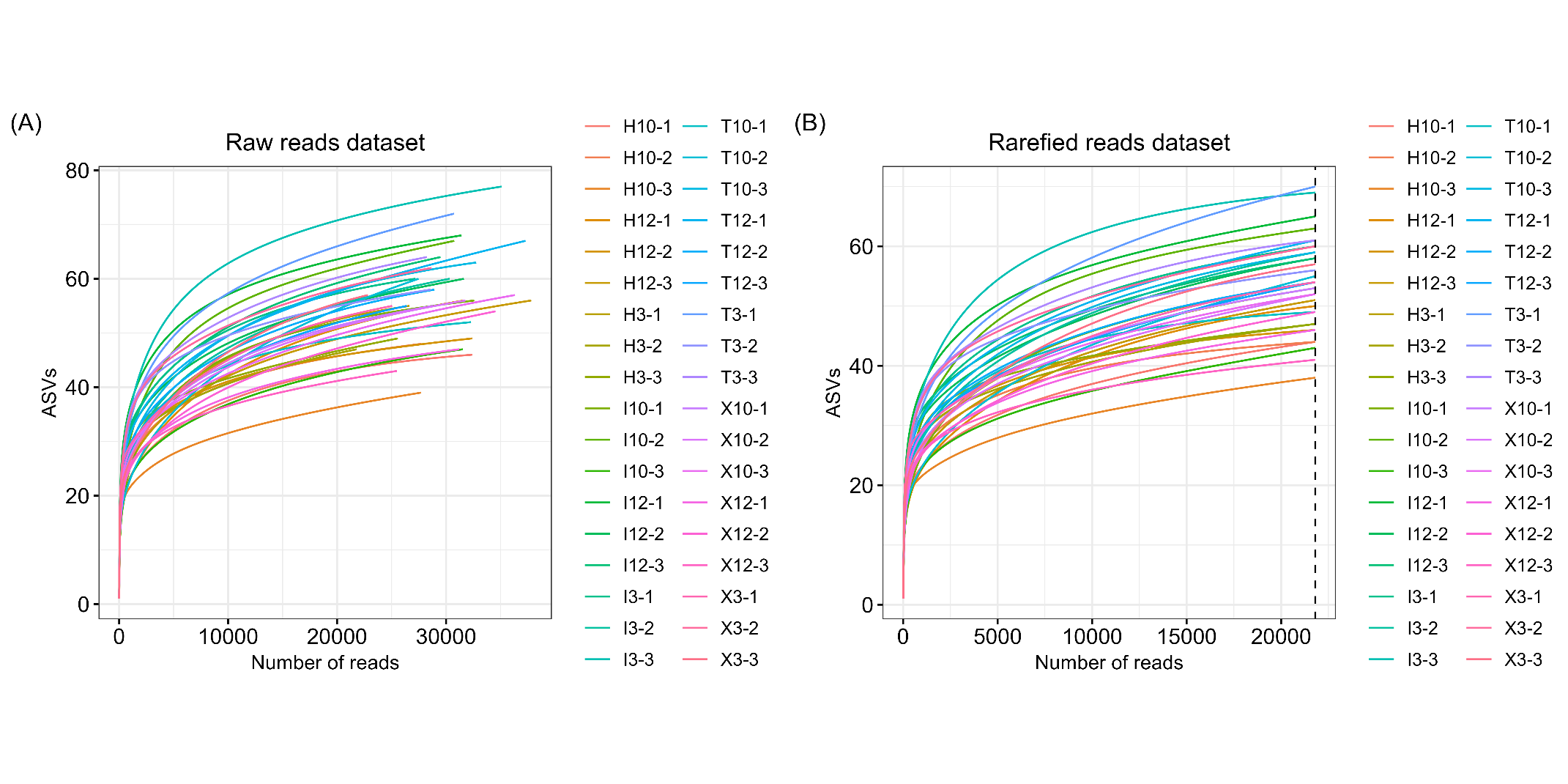
**

**Figure S1**. Rarefaction curves of the microbiota in the hindgut of the Japanese honey bees (*Apis cerana japonica*) before (A) and after (B) setting the minimal sequence read (21,808) from the raw read dataset. The black dotted line in panel (B) shows 21,808 leads as the rarefaction point. The letter in each sample indicates the colony ID, and the number indicates the month of sample collection.
